# Sustained effects of the INFORM cluster randomized trial: an observational post-intervention study

**DOI:** 10.1186/s13012-021-01151-x

**Published:** 2021-08-23

**Authors:** Matthias Hoben, Liane R. Ginsburg, Peter G. Norton, Malcolm B. Doupe, Whitney B. Berta, James W. Dearing, Janice M. Keefe, Carole A. Estabrooks

**Affiliations:** 1 Faculty of Nursing, University of Alberta, 11405 87 Avenue, Edmonton, AB T6G 1C9 Canada; 2 School of Health Policy & Management, Faculty of Health, York University, Toronto, Ontario M3J 1P3 Canada; 3 Cumming School of Medicine, University of Calgary, Calgary, Alberta T2N 4N1 Canada; 4 Department of Community Health Sciences, Rady Faculty of Health Sciences, Max Rady College of Medicine, University of Manitoba, Winnipeg, Manitoba R3E 3P5 Canada; 5 Institute of Health Policy, Management & Evaluation, Dalla Lana School of Public Health, University of Toronto, Toronto, Ontario M5T 3M6 Canada; 6 Department of Communication, College of Communication Arts and Sciences, Michigan State University, East Lansing, MI 48824 USA; 7 Department of Family Studies & Gerontology, Mount Saint Vincent University, Halifax, Nova Scotia B3M 2J6 Canada

## Abstract

**Background:**

Numerous studies have examined the efficacy and effectiveness of health services interventions. However, much less research is available on the sustainability of study outcomes. The purpose of this study was to assess the lasting benefits of INFORM (Improving Nursing Home Care Through Feedback On perfoRMance data) and associated factors 2.5 years after removal of study supports. INFORM was a complex, theory-based, three-arm, parallel cluster-randomized trial. In 2015–2016, we successfully implemented two theory-based feedback strategies (compared to a simple feedback approach) to increase nursing home (NH) care aides’ involvement in formal communications about resident care.

**Methods:**

Sustainability analyses included 51 Western Canadian NHs that had been randomly allocated to a simple and two assisted feedback interventions in INFORM. We measured care aide involvement in formal interactions (e.g., resident rounds, family conferences) and other study outcomes at baseline (T1, 09/2014-05/2015), post-intervention (T2, 01/2017-12/2017), and long-term follow-up (T3, 06/2019–03/2020). Using repeated measures, hierarchical mixed models, adjusted for care aide, care unit, and facility variables, we assess sustainability and associated factors: organizational context (leadership, culture, evaluation) and fidelity of the original INFORM intervention.

**Results:**

We analyzed data from 18 NHs (46 units, 529 care aides) in simple feedback, 19 NHs (60 units, 731 care aides) in basic assisted feedback, and 14 homes (41 units, 537 care aides) in enhanced assisted feedback. T2 (post-intervention) scores remained stable at T3 in the two enhanced feedback arms, indicating sustainability. In the simple feedback group, where scores were had remained lower than in the enhanced groups during the intervention, T3 scores rose to the level of the two enhanced feedback groups. Better culture (*β* = 0.099, 95% confidence interval [CI] 0.005; 0.192), evaluation (*β* = 0.273, 95% CI 0.196; 0.351), and fidelity enactment (*β* = 0.290, 95% CI 0.196; 0.384) increased care aide involvement in formal interactions at T3.

**Conclusions:**

Theory-informed feedback provides long-lasting improvement in care aides’ involvement in formal communications about resident care. Greater intervention intensity neither implies greater effectiveness nor sustainability. Modifiable context elements and fidelity enactment during the intervention period may facilitate sustained improvement, warranting further study—as does possible post-intervention spread of our intervention to simple feedback homes.

Contributions to the literature

- This is one of the few studies examining the sustainability (lasting benefits) of a successful intervention.
- Theory-based feedback not only is effective during the intervention period, but leads to lasting benefits 2.5 years after removal of intervention supports.
- Better work culture, evaluation processes, and fidelity enactment support lasting benefits of an intervention—regardless of the intervention intensity.

## Background

The design and evaluation of health services interventions is key to improving quality of healthcare and the patient experience. While numerous studies have examined the efficacy and effectiveness of health services interventions, much less attention has been paid to the sustainability of outcomes [1–3]. Failure to sustain intervention outcomes of effective interventions significantly limits the potential benefits of intervention investment. When researchers withdraw intervention supports, intervention activities and improved outcomes of successful interventions regularly decrease [4, 5], highlighting the need for post-intervention studies on intervention sustainability. Sustainability becomes increasingly challenging with increasing intervention complexity [6–8]—i.e., with an increasing number of intervention components interacting in complex ways, requiring multiple staff, often affecting multiple outcomes [9, 10]. According to a 2020 systematic review [11], little research has been published to date on the sustainability of complex interventions. This study responds to calls for research that examines the sustainability stage of successful interventions [5, 12] and contributes important knowledge on modifiable factors associated with sustainability of evidence-based interventions in health care settings [2].

### Study objectives

Outcomes of interest vary depending on whether a study addresses intervention use, the effects of an intervention for those people it is designed to help, or both as in hybrid study designs. In the present study, our objectives are:

1. To examine the sustainability of the primary study outcome of a successful health services trial—INFORM (Improving Nursing Home Care Through Feedback On perfoRMance data) [13–15] in each of the 3 study arms. The primary outcome in INFORM was care aide involvement in formal communications about resident care.
2. To examine the extent to which the higher intervention intensity (study arm), better fidelity of initial intervention implementation, and key contextual variables (better leadership, work culture and feedback activities [evaluation]) predict higher sustainability of the primary INFORM study outcome.

### Sustainability: definition and state of research

Reviews of sustainability research continue to identify the need for conceptual clarity and clear and consistent definitions of the construct [4, 6, 16]. A 2012 systematic review looking at the sustainability of new programs/interventions found that 65% of 125 included studies did not define sustainability with esoteric investigator-generated definitions provided in most of the remaining studies [5]. The concept of sustainability can refer to lasting benefits of an intervention and has been defined as the “an evidence-based intervention can deliver its intended benefits over an extended period of time after external support […] is terminated” [17] (p. 118). However, this definition is only “outcomes” focused while other conceptualizations of sustainability are broader and include the continued use of core intervention tools, processes, and behaviors [6, 18, 19]. For added clarity, recent work [1, 19] suggests distinguishing between sustainability (lasting benefits—i.e., sustaining or further improving study outcomes) and sustainment (continued enactment of intervention activities). In these broader conceptualizations, that encompass both sustainability and sustainment, adaptation as well as “institutionalization” of intervention activities have been identified as part of a dynamic sustainability process [1, 2, 19] taking place within complex systems [20]. Central to recent conceptualizations of sustainability is the recognition that many interventions interact with inner organizational contexts as well as outer contexts and are ideally adapted to fit those contexts [1, 12].

Sustainment studies are more common in the literature than sustainability studies. The 2012 systematic review cited above found that fewer than 25% of studies reported on the sustained impact of the program [5]. The reason there has been greater attention to sustainment of intervention activities may be that it is easier to measure and can occur within the timeframes of funded studies, compared to sustainability studies that examine lasting benefits several years beyond completion of an intervention. Studies that have examined sustainability suggest that it remains elusive [21–24], even for highly implementable prescribing practices such as use of aspirin, beta-blockers, and ACE inhibitors post AMI [25]. More common in the literature is evidence of challenges with sustainability (e.g., [8, 26–28]).

### Determinants of intervention sustainability

There are many models that identify and categorize determinants of intervention sustainability in terms of multi-level contexts internal and external to host organizations [1, 29], a theme that has long been prevalent in technology transfer and knowledge utilization literatures as “mutual adaptation” [30, 31]. Prominent models of diffusion especially in terms of the importance of compatibility of an innovation with user context [2] and sustainability [32, 33] identify aspects of the intervention (e.g., adaptability), the micro and macro context, aspects of the implementation process, as well as readiness of/fit with the organization as important determinants of sustainability. Empirical work that comprehensively tests sustainability frameworks, however, remains limited [32]. There has been some empirical work examining certain factors that influence sustainability of complex interventions [7] and large scale QI programs [34]. A recent systematic review of 32 mostly qualitative studies identified contextual variables, such as role accountability, leadership, and organizational support as the main facilitators influencing sustainability of hospital-based interventions [26]. Other recent reviews identified aspects of organizational context and capacity as important determinants of sustainability but highlight the need for more rigorous empirical work in this area [5, 32].

Additional research is necessary, particularly given the evolution of sustainability models which now reflect ecological challenges to intervention-context fit [1]. For instance, there is a need to understand whether key contextual and other determinants of sustainability hold across a range of settings and intervention types [32] and how determinants may interact to influence sustainability [5]. In addition, we found almost no studies regarding the determinants of sustainability in complex versus simple interventions. Finally, while the need for additional research on fidelity (in relation to adaptation) of sustained intervention actions is well described in the literature, we found little empirical research assessing whether fidelity of an *original* health services intervention may or may not predict sustainability. Fidelity is “the degree to which an intervention is implemented as it is prescribed in the original protocol.” [17] (p. 120).

### The INFORM trial

INFORM was a complex, pragmatic, three-arm, cluster-randomized trial designed to increase involvement of unregulated care aides in formal team communications about resident care in nursing homes. Care aides (personal support workers, nursing assistants) provide up to 90% of direct care to nursing home residents [35–38]. However, their intimate knowledge of resident care needs [39] often remains tacit rather than shared as they are rarely included in formal care decision making processes—leading to communication breakdowns and missed care [40]. While care aide involvement in formal team communications about resident care was our primary study outcome, INFORM is designed to be tailorable to address any of a wide variety of possible outcomes (e.g., care staff quality of work-life, leadership practices, or resident outcomes). We published the methods of INFORM in a trial protocol [13] and subsequently published results on INFORM’s effectiveness [14] and the impact of intervention fidelity on intervention effectiveness [15]. This study addresses an important additional aim outlined in our trial protocol [13]: to assess longer-term effects of INFORM.

INFORM was an audit and feedback intervention based on goal setting theory, designed to improve performance. We purposefully designed INFORM based on factors that positively influence implementation and sustainability, [41] including intervention attributes [42] (e.g., relative advantage, trialability or observability) and contextual elements [3, 5, 32] (e.g., engaging supportive leaders in the design and implementation of INFORM). We compared (1) a simple feedback approach to (2) a basic and (3) an enhanced assisted feedback approach. Two hundred one care unit teams in 67 Western Canadian nursing homes participated in INFORM. Teams in all three study arms received oral and written reports regarding the level of care aide involvement in formal team communications about resident care (and other contextual variables). Teams in the basic and enhanced arms also participated in three workshops where they defined learning and performance goals for increasing care aide involvement in decisions, created action plans, defined measures of success, reported progress and challenges implementing their action plans (workshops 2 and 3), and interacted with teams from other nursing homes. All three workshops in the enhanced assisted feedback arm were 3-h face to face events. In the basic assisted feedback arm, workshops 2 and 3 were virtual 1.5-h workshops.

Results showed [14] that care aide involvement in formal communications about resident care at follow up was 0.17 points higher in both the basic (95% confidence interval [CI]: 0.03; 0.32, *p* = 0.021) and enhanced study arms (95% CI 0.01; 0.33, *p* = 0.035), compared to simple feedback—with no differences between the study arms with the highest (enhanced) and mid-level (basic) intensity. Intervention fidelity was moderate to high, and higher *fidelity enactment* was associated with larger improvements in formal team communications [15]. Fidelity enactment refers to the extent to which participants adhere to and carry out core intervention components as intended by the study team during the intervention period [43].

## Methods

### Study design

This observational sustainability study is part of TREC (Translating Research in Elder Care)—a longitudinal program of applied health services research that since 2007 has collected comprehensive data on nursing home residents, care staff, care units, and facilities [44]. To assess sustainability of INFORM outcomes, we used 3 waves of TREC data: (1) pre-INFORM baseline (T1, 09/2014–05/2015), (2) post-INFORM (T2, 01/2017–12/2017), and (3) long-term follow-up (T3, 06/2019–03/2020)—data captured approximately 2.5 years following the end of the INFORM trial. These time periods constitute the waves of TREC data collection. The objective of consistent intervals of data collection is not always possible as it is contingent on the TREC research team’s resources (funding, researcher capacity) as well as the facilities capacity (sufficient staffing and no competing projects).

### Study setting and sample

**Fig. 1:**
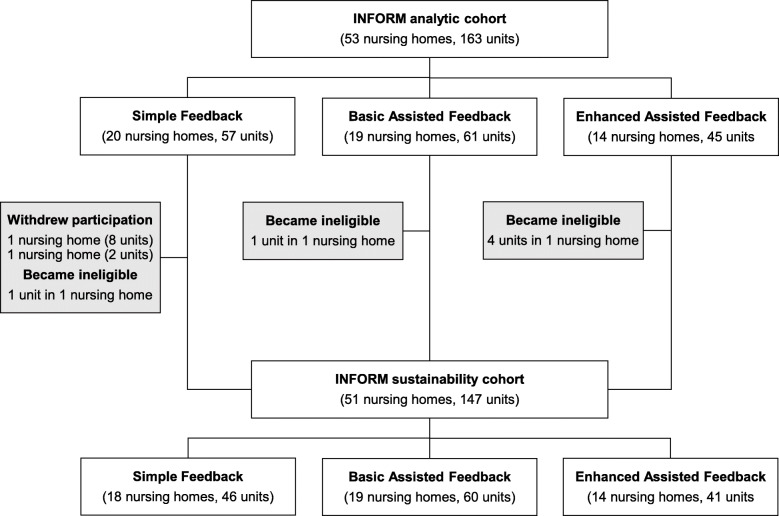
INFORM sustainability study sample

Our analytic cohort in this sustainability study included 18 facilities (46 care units) in the simple feedback arm, 19 facilities (60 care units) in the basic assisted feedback arm, and 14 facilities (41 care units) in the enhanced assisted feedback arm. Data for 16 units that were part of the initial INFORM study were not available for this sustainability study (either because a site discontinued participation in TREC or because unit configuration changed, details in Fig. 1).

### Primary study outcome

Table 1 summarizes our study outcomes. Formal interactions, the primary study outcome, is taken from the well-validated Alberta Context Tool (ACT) [45, 46] that assesses 10 modifiable features of nursing home care unit work environments. Formal interactions is a self-reported measure of care aides’ participation in 4 types of formal meetings about resident care: team meetings about residents, family conferences, change of shift reports, and continuing education outside of the care aide’s facility. Each item is rated on a 5-point scale (never–almost always).

**Table 1:** Primary and secondary study outcomes and measures

Outcome	Data source	Measure
**Primary study outcome**		
Formal interactions	ACT, embedded within the TREC care aide survey	Four items (rated from 1 = never to 5 = almost always) asking care aides how often, in the last typical month, they participated in the following: team meetings about residents, family conferences, change-of-shift reports, and continuing education (conferences, courses) outside their nursing home. A sum score (0–4, higher is better) is created by recoding each item (1 and 2 to 0; 3 to 0.5; 4 and 5 to 1) and summing recoded values.
**Predictors of sustainability**		
Leadership	ACT, embedded within the TREC care aide survey	Six items (rated from 1 = strongly disagree to 5 = strongly agree) asking care aides to rate collaborative leadership qualities of the person or group of persons they report to most of the time. An overall score is derived by averaging the score of the six items.
Culture	ACT, embedded within the TREC care aide survey	Six items (rated from 1 = strongly disagree to 5 = strongly agree) asking care aides whether they think they are part of a supportive work culture. An overall score is derived by averaging the score of the six items.
Evaluation	ACT, embedded within the TREC care aide survey	Six items (rated from 1 = strongly disagree to 5 = strongly agree) asking care aides whether they are involved in the process of using data to assess team performance and to achieve outcomes in their organization or on their care unit. An overall score is derived by averaging the score of the six items.
INFORM fidelity enactment	INFORM process evaluation toolkit	One item asking facilitators of INFORM intervention workshops to rate the level of fidelity enactment of each care unit (1 = very low to 5 = very high) after the last intervention workshop. The item score (1–5, higher is better) is used in the analyses.
**Model covariates**		
Care aide sex	Care aide demographics, embedded within the TREC care aide survey	Dichotomous item (male or female).
Care aide age	Care aide demographics, embedded within the TREC care aide survey	Categorical variable including the categories < 25 years, 25–34 years, 35–44 years, 45–54 years, > 54 years.
Care aide first language	Care aide demographics, embedded within the TREC care aide survey	Categorical variable including the categories English, Filipino, Tagalog, and other (and in case of other care aides are asked to specify the other language).
Care unit staffing	ASSiST measure, embedded within the TREC care unit survey	Average daily number of care aides, licensed practical nurses and registered nurses that are scheduled on weekdays and weekends on the unit. Care hours per resident day for each provider group and in total are calculated based on a validated algorithm.
Facility location	Facility characteristics, embedded within the TREC facility survey	Categorical variable including the categories Calgary Zone, Edmonton Zone, Fraser Health Region and Interior Health region
Facility size	Facility characteristics, embedded within the TREC facility survey	Categorical variable including the categories small (< 80 beds), medium (80–120 beds), and large (> 120 beds)
Facility ownership model	Facility characteristics, embedded within the TREC facility survey	Categorical variable, including the categories public, not-for-profit; voluntary, not-for-profit; private, for-profit

*ACT* Alberta Context Tool; *ASSiST* A Scheduled Shifts Staffing; *TREC* Translating Research in Elder Care

### Independent variables and covariates

Consistent with the Promoting Action on Research Implementation in Health Services (PARiHS) framework [47–49], we included the following contextual variables in our analysis (also part of the ACT): leadership (6 items rating care aides’ perception of transformational leadership of the person they report to most of the time, 5-point agreement scale, strongly disagree–strongly agree), culture (6 items rating care aides’ perception of the supportive work culture, 5-point agreement scale, strongly disagree–strongly agree), and evaluation (6 items rating care aides’ participation in data-based feedback and performance improvement activities, 5-point agreement scale, strongly disagree–strongly agree) (Table 1). We also included fidelity enactment, measured using a 5-point scale used in the initial INFORM study at the close of the intervention [15]. We adjusted our sustainability models for the same variables that we used to adjust models in our effectiveness study [14]: care aide age, sex, and first language [44]; care unit staffing [50]; and facility region, size, and ownership (Table 1).

### Statistical analyses

Using SAS® 9.4, and following analytic methods described in our intervention effectiveness paper [14], we first descriptively compared our primary study outcome (care aide reported involvement in formal team communications about resident care—formal interactions) and covariates by study arm and time of data collection. To compare trajectories of the primary study outcome by study arm and time of data collection (objective 1), we estimated adjusted least square means and mean differences of these outcomes, using repeated measures mixed effects regression models with random intercepts for care unit and facility levels, and a random effect for care aides responding to our survey repeatedly. We added study arm and time of data collection as categorical variables, included an interaction term between study arm and time of data collection, and adjusted for facility variables (region, owner-operator model, size), care unit staffing (total care hours per resident day and percentage of total hours per resident day provided by care aides), and care aide characteristics (sex, age, English as first language [yes/no]).

Finally, to assess the impact of organizational context and fidelity enactment on the sustainability of our primary study outcome (objective 2), we specified two mixed effects regression models with formal interactions at T3 as the dependent variable. To the first model, we added leadership, culture, and evaluation at T3 and adjusted for variables that, based on our initial models, were predictive of formal interactions: study arm, region, care aide’s sex, and care aide’s first language. We also adjusted for the unit aggregated T2 formal interactions score (to account for differences in formal interactions pre-sustainability measurement). In addition, we ran the same model with fidelity enactment added (model 2). Since this outcome was only available in the assisted feedback arms, we excluded the simple feedback sample from this model. To assess whether the strength of the effects between our four main independent variables (leadership, culture, evaluation, and—for model 2—fidelity enactment) differed by study arm, we added interaction terms between each of these four variables and study arm. None of interaction terms improved model fit (based on the Akaike information criterion (AIC) and the Bayesian information criterion (BIC) [51]) or had *p* values < 0.05. Despite the common practice to accept *p* values of up to 0.25 as statistically significant (i.e., raising the type 1 error rate), we decided—in line with the recommendation of a recent simulation study [52]—not to follow this practice since this increases the risk of including spurious interaction terms. Therefore, we did not include any interaction terms in the final models.

The amount of missing data in our data set was minimal. Leadership was missing in 35 of 11,988 (0.29%) of the records, culture was missing in 13 (0.11%) of the records, and evaluation was missing in 29 (0.24%) of the records. Data were missing completely at random as per Little’s MCAR test. No responses were missing for any of our other variables. Therefore, we deleted records with missing data listwise.

### Sensitivity analyses

We conducted sensitivity analyses to address the question whether the trends in formal interactions that we found in our analyses may constitute an issue of regression to the mean. We compared trends in formal interactions in our three study arms to those in 12 TREC nursing homes (data from 544 care aides on 44 care units) located in the province of Manitoba, a province in which we had not carried out any INFORM activities. Therefore, the Manitoba sample can be seen as a natural control group.

## Results

Table 2 includes facility, care unit, and care aide characteristics by study arm and time of data collection. Based on our descriptive statistics, total staffing hours per resident day, the proportion of care aide hours among total care staffing hours, and the proportion of care aides whose first language was not English seemed to differ between study arms (at all three points in time) and changed over time within each study arm. Care aide age and sex did not differ substantially between study arms and were stable over time. The Manitoba sample did not differ substantially from our Alberta and British Columbia samples.

**Table 2:** Facility, care unit, and care aide characteristics at by time of data collection and study arm

	SF	BAF	EAF	Winnipeg	SF	BAF	EAF	Winnipeg	SF	BAF	EAF	Winnipeg
**Facility sample**												
Number of facilities	18	19	14	12	—	—	—	—	—	—	—	—
Region												
Calgary	4 (22.2%)	4 (21.1%)	3 (21.4%)	—	—	—	—	—	—	—	—	—
Edmonton	4 (22.2%)	5 (26.3%)	3 (21.4%)	—	—	—	—	—	—	—	—	—
Fraser Health	6 (33.3%)	6 (31.6%)	6 (42.9%)	—	—	—	—	—	—	—	—	—
Interior Health	4 (22.2%)	4 (21.1%)	2 (14.3%)	—	—	—	—	—	—	—	—	—
Size												
Small (< 80 beds)	6 (33.3%)	6 (31.6%)	3 (21.4%)	2 (16.7%)	—	—	—	—	—	—	—	—
Medium (80–120 beds)	4 (22.2%)	7 (36.8%)	5 (35.7%)	4 (33.3%)	—	—	—	—	—	—	—	—
Large (> 120 beds)	8 (44.4%)	6 (31.6%)	6 (42.9%)	6 (50.0%)	—	—	—	—	—	—	—	—
Ownership												
Public, not-for-profit	5 (27.8%)	5 (26.3%)	3 (21.4%)	2 (16.7%)	—	—	—	—	—	—	—	—
Voluntary, not-for-profit	5 (27.8%)	6 (31.6%)	3 (21.4%)	6 (50.0%)	—	—	—	—	—	—	—	—
Private, for-profit	8 (44.4%)	8 (42.1%)	8 (57.1%)	4 (33.3%)	—	—	—	—	—	—	—	—
**Care unit sample**												
Number of care units	46	60	41	44	—	—	—	—	—	—	—	—
Care unit staffing (M ± SD)												
Total care hours per resident day	3.4 ± 1.5	3.2 ± 1.0	3.2 ± 0.8	2.9 ± 0.5	3.0 ± 0.7	2.5 ± 1.2	2.7 ± 0.8	3.0 ± 0.5	2.7 ± 0.5	2.6 ± 1.1	2.9 ± 0.6	3.0 ± 0.5
Proportion of care aide hours among total care hours per resident day	63.7 ± 13.0	71.9 ± 10.0	73.0 ± 8.4	70.1 ± 5.7	69.5 ± 9.1	68.9 ± 10.5	73.4 ± 9.8	66.8 ± 5.1	69.2 ± 6.6	68.1 ± 11.2	67.5 ± 8.0	65.1 ± 8.6
**Care aide sample**												
Number of care aides	621	824	566	544	624	789	543	586	533	737	538	594
Females	560 (90.2%)	753 (91.4%)	505 (89.2%)	474 (87.1%)	554 (88.8%)	728 (92.4%)	494 (91.3%)	513 (87.5%)	489 (91.7%)	679 (92.1%)	487 (90.5%)	502 (84.0%)
Age category												
< 25 years	17 (2.7%)	13 (1.6%)	30 (5.3%)	23 (4.2%)	25 (4.0%)	14 (1.8%)	32 (5.9%)	10 (1.7%)	11 (2.1%)	25 (3.4%)	14 (2.6%)	8 (1.3%)
25–34 years	130 (20.9%)	117 (14.2%)	98 (17.3%)	82 (15.1%)	102 (16.4%)	112 (14.2%)	97 (17.9%)	83 (14.2%)	88 (16.5%)	96 (13.0%)	101 (18.8%)	83 (13.9%)
35–44 years	184 (29.6%)	256 (31.1%)	163 (28.8%)	130 (23.9%)	167 (26.8%)	232 (29.4%)	134 (24.7%	155 (26.5%)	143 (26.8%)	195 (26.5%)	130 (24.2%)	147 (24.5%)
45–54 years	169 (27.2%)	259 (31.4%)	170 (30.0%)	176 (32.4%)	191 (30.6%)	254 (32.2%)	172 (31.7%)	175 (29.9%)	166 (31.1%)	242 (32.8%)	167 (31.0%)	190 (31.7%)
> 54 years	121 (19.5%)	179 (21.7%)	105 (18.6%)	133 (24.5%)	139 (22.3%)	177 (22.4%)	108 (19.9%)	163 (27.8%)	125 (23.5%)	179 (24.3%)	126 (23.4%)	171 (28.6%)
English as second language	375 (60.4%)	569 (69.1%)	357 (63.1%)	362 (66.5%)	382 (61.2%)	567 (71.9%)	384 (70.7%)	433 (73.9%)	351 (65.9%)	554 (75.2%)	370 (68.8%)	450 (75.1%)

—, outcome has not changed over time; *BAF* basic assisted feedback; *EAF* enhanced assisted feedback; *SF* simple feedback

**Fig. 2:**
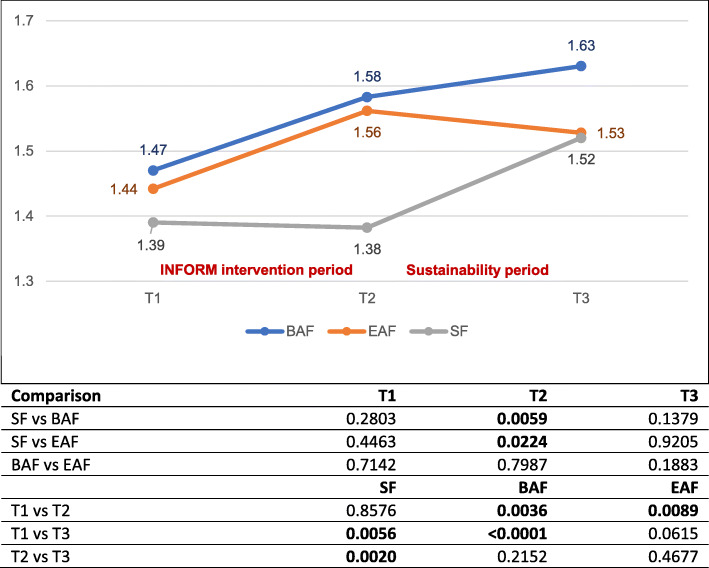
Adjusted formal interactions scores by study arm and time of assessment. Numbers presented in the figure are adjusted least square means based on repeated measures mixed effects regression models. Numbers in the table are *p* values of adjusted mean differences based on repeated measures mixed effects regression models. BAF, basic assisted feedback; EAF, enhanced assisted feedback; SF, simple feedback

Our adjusted analyses (Fig. 2) demonstrate that at T3 (2.5 years after the intervention delivery had ended), care units in both assisted feedback groups had sustained the T2 (post-intervention) gains in care aide involvement in formal communications about resident care, indicating sustainability. While there seems to be a T2-T3 downward trend in the enhanced assisted feedback arm and a T2-T3 upward trend in the basic assisted feedback arm, neither of these group-specific trends are statistically significant. Notably, involvement of care aides in formal communications about resident care did not increase during the intervention period in the simple feedback group. However, it increased significantly at T3, rising to the levels seen in the two assisted feedback arms.

**Fig. 3:**
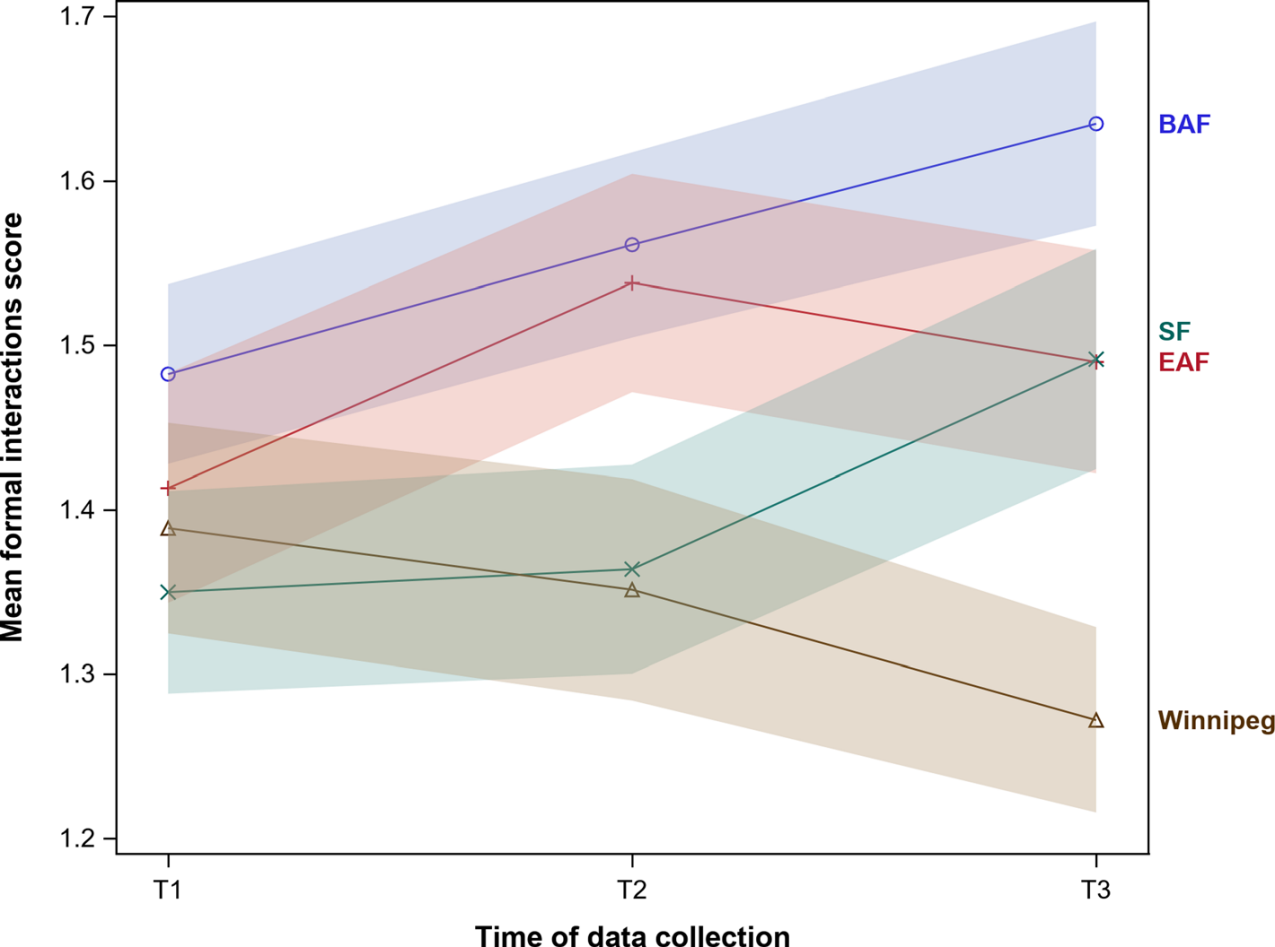
Unadjusted formal interactions scores by study arm and time of assessment, including the Winnipeg sample of nursing homes (bands are 95% confidence intervals). BAF, basic assisted feedback; EAF, enhanced assisted feedback; SF, simple feedback

Our sensitivity analyses (Fig. 3) illustrate that in the Manitoba sample formal interactions scores followed a consistent downward trend that differs substantially from the trends seen in the three INFORM study arms.

As per Table 3, evaluation (feedback of performance data) at T3 was associated statistically significantly with higher involvement of care aides in formal communications about resident care at T3 in both models. Higher leadership scores (i.e., care aide ratings of the leadership of the persons they report to—primarily nurses) were not associated with care aide involvement in formal communications about resident care at T3. A more supportive work culture was associated with higher involvement of care aides in formal communications about resident care at T3 in model 1 (that included the simple feedback sample) but not in model 2. However, in model 2, better fidelity enactment at T2 predicted higher T3 involvement of care aides in formal communications about resident care. As noted, none of our interaction terms (fidelity by study arm and each of leadership, culture, evaluation by study arm) were statistically significant (*p* values consistently > 0.2) nor did they improve model fit and we therefore did not include any interaction terms in our final model. None of the other covariates were associated with care aide involvement in formal team communications about resident care at T3.

**Table 3:** Association of organizational context (leadership, culture, evaluation) and fidelity enactment with formal interactions, based on adjusted mixed effects regressions

	Model 1 (SF included)					Model 2 (SF excluded)				
	Est.	SE	LCI	UCI	P	Est.	SE	LCI	UCI	P
Intercept	0.317	0.220	− 0.127	0.760	0.1578	0.152	0.287	− 0.431	0.735	0.5972
Organizational context										
ACT leadership	− 0.029	0.041	− 0.110	0.051	0.4734	− 0.006	0.050	− 0.104	0.093	0.9083
ACT culture	0.099	0.048	0.005	0.192	**0.0389**	0.066	0.058	− 0.047	0.180	0.2518
ACT evaluation	0.273	0.040	0.196	0.351	**<.0001**	0.290	0.048	0.196	0.384	**< .0001**
Fidelity enactment	--	--	--	--	--	0.047	0.021	0.005	0.089	**0.0277**
Study arm*(ref. = SF)*										
BAF	0.071	0.082	− 0.091	0.232	0.3897	0.055	0.089	− 0.120	0.229	0.5392
EAF	0.059	0.086	− 0.110	0.228	0.4914	--	--	--	--	--
Unit-level wave 4 FI score	− 0.021	0.088	− 0.194	0.152	0.8116	− 0.044	0.108	− 0.257	0.168	0.6825
Region*(ref. = Interior)*										
Calgary	0.108	0.111	− 0.109	0.325	0.3288	0.205	0.147	− 0.083	0.494	0.1625
Edmonton	− 0.046	0.110	− 0.263	0.170	0.6743	0.040	0.144	− 0.243	0.323	0.7817
Fraser	− 0.126	0.105	− 0.332	0.080	0.2293	− 0.089	0.141	− 0.365	0.187	0.5280
Care aide is female	− 0.107	0.067	− 0.238	0.025	0.1123	− 0.112	0.081	− 0.271	0.046	0.1653
Care aide first language not English	0.017	0.046	− 0.073	0.107	0.7163	0.059	0.056	− 0.050	0.169	0.2894

*ACT* Alberta Context Tool; *BAF* basic assisted feedback; *EAF* enhanced assisted feedback; *Est.* estimate; *FI* formal interactions; *LCI* lower 95% confidence interval limit of estimate; *SE* standard error; *SF* simple feedback; *UCI* upper 95% confidence interval limit of estimate

## Discussion

This study examined sustained effects of the INFORM intervention as well as the association of fidelity enactment and key variables of organizational context with sustained involvement of care aides in formal communications about resident care (formal interactions). Our findings suggest sustained benefits of the INFORM intervention 2.5 years after the research team had finalized the delivery of the intervention—signified by the lack of a statistically significant difference in formal interactions scores between T2 and T3 in both assisted feedback groups. Like at T2, formal interactions scores in the two assisted feedback groups did not differ at T3. Notably, formal interaction scores did not increase in the simple feedback arm during the intervention (signified by the lack of a statistically significant difference in formal interactions scores between T1 and T2), but rose to the levels of the assisted feedback arms at T3 after the end of the INFORM study. Higher fidelity enactment during the intervention period, more supportive work culture, and more feedback activities (evaluation) with a care unit were associated with higher formal interaction scores at T3, regardless of the intervention intensity (study arm allocation).

Our study is one of the few that has examined the degree to which the effects of a complex intervention can be sustained in a complex care setting, such as nursing homes. TREC’s comprehensive database of longitudinal data is one of the main reasons why we could conduct this work and provide these insights—highlighting the critical need for more longitudinal studies of intervention effectiveness and sustainability that cover time periods of 5 years or more.

Our finding that higher fidelity enactment during the intervention period was associated with sustained intervention benefits is noteworthy. To the best of our knowledge, this is the first study to examine this association. As noted, fidelity enactment refers to the extent to which participants adhere to and carry out core intervention components as intended during the intervention period [43]. Core components of the INFORM intervention include (1) managers and care teams setting learning and performance goals, (2) managers engaging with care teams to work towards goal achievement, and (3) measuring success towards these goals. While fidelity enactment is critical for intervention success, its role for intervention sustainability is poorly understood as reported in contemporary empirical research. The dynamic sustainability framework (DSF) suggests that intervention approaches that emphasize adaptation (adjusting and refining the intervention to fit the local context) sustain an intervention more successfully than approaches that emphasize fidelity to an original protocol [1]. Future research is needed to better understand how and why care teams that adhere more closely to intervention protocols are more successful in sustaining intervention benefits and what role adaptation plays in these processes. Additionally, related research is needed on the extent to which successful care teams are able to adapt interventions to their needs, under what circumstances these adaptations violate intervention fidelity, and attain outcomes that are superior to those originally reported in clinical trials, and how different ways of adapting the intervention affect sustainability [21, 60].

Our finding that evaluation (feedback of performance data to care teams) was associated with sustained intervention success is in line with previous studies [3, 5, 32]. Evaluation is part of what the DSF labels as information systems [1]. Evaluation activities—i.e., consistent feedback on a team’s performance and discussion of possible solutions—facilitate rapid learning and real-time problem-solving among care teams and integrate team members in the generation, rather than just the application of knowledge. The DSF assumes that an organization that develops this kind of a culture will be more successful in sustaining an intervention by improving the fit of an intervention over time.

Our finding that leadership was not associated with sustainability is not consistent with available reviews [3, 5, 32], nor with the DSF (where this construct is labeled supervision) [1]. For example, Hailemariam et al. [3] found 12 studies suggesting an association of leadership with intervention sustainability. However, from these studies, it is largely unclear what type of leadership style was associated with sustainability. For example, in their systematic review, Hailemariam et al. [3] found that “organizational leadership” was consistently associated with sustainment of evidence-based practices, but the authors provide no further details on the leadership styles measured. The ACT leadership scale [45, 46] that we used in our survey measures transformational leadership, which is only one of many leadership styles that may have been measured by other studies. More research is needed to determine whether this leadership style is associated with sustainability of study outcomes. Furthermore, the ACT leadership scale asks care aides to rate leadership characteristics of the person (or group of persons) to whom they report most of the time. In nursing home settings, these individuals are generally registered nurses or licensed practical nurses [53]. Therefore, the leadership scale we used measures care aides’ rating of nurses’ leadership, not their rating of facility-level or unit-level formal leaders/supervisors (organizational leadership). Care aides rate, for example, whether nurses regularly ask them for feedback even if it is difficult to hear, actively mentor or coach them, or focus on success rather than failure. This rating of nurses’ leadership may not be reflective of the managers’ leadership—and managers, not nurses, are the ones enabling, encouraging, and/or requiring care aides to participate in formal team meetings about resident care. Furthermore, the leadership skills listed above are not necessarily specific to enabling and encouraging a care aide to participate in formal meetings about resident care. A specific leadership skill would be, for example, a manager’s ability to recognize and address the care aide’s specific needs (such as lack of confidence to participate in such meetings, inability to envision what to say and how to best contribute, concern to neglect residents while participating, etc.).

Our finding that culture was associated with sustainability is in line with the DSF [1], but is not consistent with available reviews [3, 5, 32]. Culture has been identified as a factor associated with sustainability in only a small number of studies [3, 5, 32]. In their review, Stirman et al. [5] highlight that the small number of studies empirically supporting culture to be a key factor for intervention sustainability stands in contrast with the extent to which culture is discussed as an important factor in the implementation and sustainability literature. This most likely has to do with how culture is operationalized and measured. For instance, qualitative studies suggest that local unit-level processes and interactions that may reflect a particular culture or leadership style are more likely to support sustainability than the overall culture of an organization (which is commonly measured in quantitative sustainability studies) [5]. The ACT culture scale [45, 46] we used in this study asks care aides about particular interactions and processes (e.g., whether they are members of a supportive work group or whether their immediate team works to provide what residents need).

We found that the factors influencing sustainability were independent of whether the intervention participants were exposed to the more versus the less intense study arm. We even saw a slightly lower success and sustainability in our most intense study arm, compared to the less intense study arm (although not statistically significant). It is possible that our enhanced feedback was too onerous for participants (meeting face-to-face for half-day workshops, versus 1.5-h video conferences in the basic assisted arm), limiting short- and long-term benefits. An alternative explanation is that the minimum “dose” of feedback required to facilitate sustainability was achieved with the less intense study arm. This finding contrasts with clinical studies reporting that higher intervention intensity supports better intervention success and sustainability [54–56]. Shelton et al. [32] highlight the important research gap related to whether sustainability depends on the type and intensity of the intervention. Little work is available on how to determine the optimal intensity of an intervention, and a particular gap exists regarding the influence of intervention intensity on the sustainability of highly complex health services interventions. Our study contributes in important ways to addressing this knowledge gap. However, more research is needed on whether the factors we identified applying a complex intervention in nursing homes are consistent or different in other healthcare settings or when applying different types of interventions.

We found that homes in the simple feedback arm saw a substantial increase in their formal interaction scores in the post-intervention period. In contrast to nursing homes in the assisted feedback arms, simple feedback homes did not receive major intervention components, such as goal setting and support workshops, support by the study team during intervention workshops to set goals and complete an action plan, peer-to-peer support by teams from other nursing homes, etc. However, we systematically fed back INFORM findings to facilities and decision makers as part of our routine feedback activities after the T2 data collections (i.e., after the INFORM intervention period). It is also possible that TREC’s relationships with key stakeholders over 10–15 years (regional and provincial decision makers, nursing home owner-operators and care teams), their systematic involvement with INFORM, and our longitudinal work with nursing homes in our cohort may have played an important role. In fact, many of the key stakeholders in our study know each other and actively collaborate on a regular basis. For example, health region decision makes hold regular meetings with nursing home administrators in their region, giving decision makers the opportunity to interact with facilities and allowing administrators to interact and exchange information across nursing homes. Often, nursing home administrators or directors of care oversee more than one facility. Administrators of facilities operated by the same owner regularly meet and collaborate on improvement activities. While this is not a definite explanation of the increased informal interaction scores we found in non-intervention facilities after the end of our intervention, it is a plausible explanation. However, the exact reasons of this surprising finding are an interesting and hard to understand question. Pending resources, we are hoping to conduct focus groups with decision makers and key stakeholders in our facilities to further explore this question.

### Study strengths and limitations

Our study has important strengths. It is one of the few studies available that assesses longer-term effects of a rigorous, complex health services intervention. We used comprehensive longitudinal data, collected using validated surveys from a large, representative sample of nursing homes and care aides. The involvement of key system- and practice-level stakeholders aimed at improving intervention success may have contributed to sustainability—and possibly unintentional and informal spread. There are, however, some limitations to note. While TREC facilities are sampled to be representative of the nursing home population in Western Canada (using a stratified, random sampling approach), facilities that have decided to engage and stay engaged with TREC for years may be more motivated, more ready for change, and have different resource configurations than other facilities. Therefore, generalizability of our findings to non-TREC homes may be limited. Future work needs to empirically investigate the question whether and how TREC facilities are different from non-TREC facilities.

As discussed in our main trial results paper [14], the effect sizes of increased formal interactions scores are small (we found Cohen’s *d* values of less than 0.2). However, our absolute improvement in formal interactions was 6.4%, which is comparable to effect sizes found in other audit and feedback studies [57, 58]. Like at the time when the intervention ended, we still do not know whether such small increases in formal interactions are sufficient to improve resident or care staff outcomes. As pointed out by Wensing and Grol [59], effects of interventions such as INFORM are incremental in nature. Studies are rarely funded over a long enough time for these interventions to improve resident and care staff outcomes. Our plan was to assess whether formal interactions were further sustained and whether care staff and resident outcomes hat started to improve in INFORM homes at TREC’s next wave of survey data collection (at the end 0f 2021). However, the COVID-19 pandemic likely has overridden any such effect, making it impossible to answer this question.

From our study, we do not know to what extent the sustained benefits in study outcomes are due to sustainment of intervention activities in care sites (i.e., if teams that successfully enacted core components during the intervention kept doing so after the research team had stopped delivering the intervention). To advance knowledge in this area, future studies should endeavor to look at both the sustainability of outcomes *and* the sustainment of intervention actions after the end of an intervention study.

## Conclusions

Our study findings suggest that the benefits in INFORM’s primary outcome—care aide involvement in formal interactions about resident care—was sustained in the two more intense study arms, 2.5 years after intervention delivery had ended. Care teams in our least intense study arm started to increase care aide involvement in formal interactions about resident care after our trial had ended, raising the possibility of informal spread. Higher fidelity enactment during the intervention period, more supportive work culture, and more feedback activities (evaluation) within a care unit were associated with sustained care aide involvement in formal communications about resident care, regardless of the intervention intensity (study arm allocation). This study supports some of the assumptions posed by the DSF, but also raises questions—especially related to the role of intervention fidelity versus adaptation and their complex interplay.

## Data Availability

The data used for this article are housed in the secure and confidential Health Research Data Repository (HRDR) in the Faculty of Nursing at the University of Alberta (https://www.ualberta.ca/nursing/research/supports-and-services/hrdr), in accordance with the health privacy legislation of participating TREC jurisdictions. These health privacy legislations and the ethics approvals covering TREC data do not allow public sharing or removal of completely disaggregated data (resident-level records) from the HRDR, even if de-identified. The data were provided under specific data sharing agreements only for approved use by TREC within the HRDR. Where necessary, access to the HRDR to review the original source data may be granted to those who meet pre-specified criteria for confidential access, available at request from the TREC data unit manager (https://trecresearch.ca/about/people), with the consent of the original data providers and the required privacy and ethical review bodies. Statistical and anonymous aggregate data, the full dataset creation plan, and underlying analytic code associated with this paper are available from the authors upon request, understanding that the programs may rely on coding templates or macros that are unique to TREC.

## Abbreviations

INFORM: Improving Nursing Home Care Through Feedback On perfoRMance data
TREC: Translating Research in Elder Care
